# Metabolic changes during exclusive enteral nutrition in pediatric Crohn’s disease patients

**DOI:** 10.1007/s11306-022-01953-0

**Published:** 2022-11-25

**Authors:** Jair G. Marques, Tobias Schwerd, Philip Bufler, Sibylle Koletzko, Berthold Koletzko

**Affiliations:** 1 Department of Pediatrics, Dr. von Hauner Children’s Hospital, University Hospital, LMU Klinikum Munich, Munich, Germany; 2 Department of Pediatric Gastroenterology, Nephrology and Metabolic Diseases, Charité- Charité Universitätsmedizin Berlin, Corporate member of Freie Universität Berlin and Humboldt-Universität zu Berlin, Berlin, Germany; 3 Department of Pediatrics, Gastroenterology and Nutrition, School of Medicine Collegium Medicum, University of Warmia and Mazury, Olsztyn, Poland; 4 Dr. von Hauner Children’s Hospital, University Hospital, Campus Innenstadt Ludwig-Maximilians-Universität München, Lindwurmstr. 4, D-80337 Muenchen, Germany

**Keywords:** Metabolomics, Biomarkers, Crohn’s disease, Exclusive Enteral Nutrition

## Abstract

**Background and aims:**

Exclusive enteral nutrition is recommended as a first-line treatment in active pediatric Crohn’s Disease, but its mechanism of action is still not clear. We aimed to assess alterations in the metabolic profile of newly diagnosed pediatric Crohn’s Disease patients before and during exclusive enteral nutrition therapy.

**Methods:**

Plasma samples from 14 pediatric Crohn’s Disease patients before and after 3–4 weeks on exclusive enteral nutrition were analyzed using mass spectrometry. T-test, fold change and orthogonal partial least squares discriminant analysis were used for mining significant features. Correlation analysis was performed between the annotated features and the weighted pediatric Crohn’s disease activity index using Pearson r distance.

**Results:**

Among the 13 compounds which decreased during exclusive enteral nutrition, most are related to diet, while one is a bacterial metabolite, Bacteriohopane-32,33,34,35-tetrol. The phosphatidic acid metabolite PA(15:1/18:0) was significantly reduced and correlated with the weighted pediatric Crohn’s disease activity index. Lipids increased during exclusive enteral nutrition therapy included phosphatidylethanolamines; PE(24:1/24:1), PE(17:2/20:2) and one lactosylceramide; LacCer(d18:1/14:0).

**Conclusion:**

Food additives and other phytochemicals were the major metabolites, which decreased following the exclusion of a regular diet during exclusive enteral nutrition. An alteration in bacterial biomarkers may reflect changes in intestinal microbiota composition and metabolism. Thus, metabolomics provides an opportunity to characterize the molecular mechanisms of dietary factors triggering Crohn’s Disease activity, and the mechanisms of action of exclusive enteral nutrition, thereby providing the basis for the development and evaluation of improved intervention strategies for prevention and treatment.

**Supplementary Information:**

The online version contains supplementary material available at 10.1007/s11306-022-01953-0.

## Introduction

Genetic and environmental factors, including the gut microbiota, play an important role in the pathophysiology of Crohn’s disease (CD) [1]. CD has been attributed to complex mechanisms triggering an dysregulated immune response to commensal gut microbiota [2]. Exclusive enteral nutrition (EEN) is an established therapy to induce remission in pediatric CD patients with efficacy in up to 80% of individuals [3], and it is recommended as first-line therapy in European guidelines [4–6]. A meta-analysis based on 18 studies concluded that there is no significant difference in efficacy using EEN or corticosteroids treatment to induce remission in pediatric CD, however, EEN seems to be superior in promoting mucosal healing and faster reduction of PCDAI (pediatric Chrohn’s disease activity index) [7]. The benefits of using EEN in pediatric patients extend beyond promoting mucosal healing, contributing to the improvement of nutritional status [8], bone metabolism, and muscle mass [9]. However, the mechanism of EEN action is still unclear. Several studies have focused on investigating how EEN may affect the microbiome, and most report an overall decrease in microbiome diversity during EEN therapy [10–17]. However, the use of different techniques to assess taxonomic shifts and the high diversity of microorganisms present in the human microbiota and its interindividual variation generates heterogenous results, especially at taxonomic resolution lower than phylum-level [18]. In addition to changes in microbiota composition, CD-associated dysbiosis affects microbial metabolic functions. Previous research demonstrated alterations in microbial functions with a shift in genetic abundance related to oxidative stress pathways, carbohydrate metabolism and amino acid biosynthesis, which was considered more disturbed than microbiota composition shifts [19]. In another observational study, the authors reported a reduction in metabolic activity of the intestinal microbiome during enteral feeding for two weeks, based on analyzing exhaled breath and fecal samples using gas chromatography/mass spectrometry [20]. Recently, Diederen et al. reported a reduction in microbiome diversity and changes in the fecal metabolome during EEN in pediatric CD patients, with alterations in amino acids, cadaverine, trimethylamine, and bile acids levels [21].

Over the past years, untargeted metabolomics using mass spectrometry has been applied as powerful tool for identification and tracking of biomarkers which help in understanding the system biology and treatment outcomes [22]. Metabolomics have provided new insights into metabolic alterations in CD patients versus healthy subjects using both serum and fecal samples [23,24]. However, only few studies were performed on pediatric population [25] and could not detect the differences induced by EEN therapy, which is one of the points of strength of our study.

## Methods

### Plasma samples

Plasma samples from 14 pediatric CD patients (age (mean ± SD) 13.5 ± 2.2 years, 8 boys, 12 newly diagnosed), before and after 25 ± 5 days on EEN treatment were analyzed. Thirteen patients received Modulen® IBD (Nestlé Nutrition) and one patient was treated with Neocate Junior^®^ (Nutricia). Samples were obtained from a previously published study [15].

The sample preparation procedure was previously described 26. Briefly, after samples were thawed on ice, 100 µL of plasma were precipitated with the addition of 900 µL of ice-cold high-performance liquid chromatography (HPLC)-grade methanol in a 1.5 mL Eppendorf tube, vortexed and rested at -20 °C for 30 min. The tubes were then centrifuged, and the supernatant was filtered in a polytetrafluoroethylene (PTFE) 45 μm 96-well filter plate. Samples were kept at − 80 °C before analysis. Quality control (QC) samples were created by pooling aliquots from the study samples and used to create an inclusion list in the method development and to ensure reproducibility in the analysis.

### LC-QTOF-MS(/MS)

The analysis was conducted on a 1290 Infinity II HPLC system using a Poroshell 120 EC-C18 column (2.1 × 150 mm, 2.7 μm) coupled to a 6545 Q-TOF (both from Agilent, Santa Clara, CA), as previously described [26]. Briefly, 6 µL of the quality control sample were injected in triplicate in full scan (MS) acquisition mode. Data from the MS experiment was then used to create an acquisition list to be used in the auto MS/MS acquisition mode. The analysis was performed with the instrument in the 2 GHz, extended dynamic range in the negative ionization (NEG) mode using an Agilent Jet Stream (AJS) electrospray ionization (ESI) ion source. Operation parameters were set as follows: capillary voltage: − 4000 V; nozzle voltage − 500 V; nebulizer pressure: 40 psi; gas temperature: 290 °C; sheath gas flow: 12 L/min; sheath gas temperature: 380 °C; fragmentor voltage: 170 V; and skimmer voltage: 65 V. The instrument was operated using MassHunter Acquisition B.09.00 software (Agilent). Chromatographic separation was achieved in 16 min run time using a mobile phase A, water (0.1% formic acid); and B, methanol (0.1% formic acid) at a flow rate of 0.4 mL/min. Gradient elution was performed with an initial mixture of 5% B and 95% A, then increased to 60% B throughout 4 min, to 99% B at 12 min, held until 14 min, returned to 5% B at 15.1 min, and held to 16 min.

#### LC-QTOF-MS analysis

The full-scan analysis was performed in triplicate using the QC samples acquired over the range of 100 to 1050 m/z in the NEG mode to create an inclusion list to further create the auto MS/MS acquisition mode. Data were extracted using batch recursive feature extraction algorithm in MassHunter Profinder B.08.00 software (Agilent) and after evaluation exported as CEF (Cluster Exchange Format) files. The features were aligned on Mass Profiler software (Agilent) using retention time (RT) tolerance of up to 0.3 min and mass tolerance of ± 15 ppm. Features with a 100% occurrence in the replicates were used to create a target MS/MS inclusion list.

#### LC-QTOF-MS/MS analysis

Analysis of the study samples was performed using data-dependent acquisition (DDA) (auto MS/MS) acquisition mode using the inclusion list as preferred ions for fragmentation, using delta m/z of 15 ppm and delta retention time (RT) of 0.15 min. The collision cell operates with fixed collision energies of 10, 20, and 40 eV using nitrogen (N2) as the collision gas. The acquisition parameters were set as follows: acquisition mass range: 100 to 1050 m/z at 4 spectra/s in the MS, and 50–800 m/z at 3 spectra/s in the time of flight (TOF).

### Data processing and statistical analysis

Samples from 14 pediatric CD patients, from before and after at least 19 days of EEN were analyzed pairwise. Data were processed as described in our previous work [26]. Briefly, quantile normalization was applied on raw data for features filtered based on QC procedure, where features with less than 100% occurrence between the QC and with coefficient of variance higher than 25% were excluded. Principal component analysis was used to perform a QC on samples to exclude any outlier by visual inspection. Identification of features was performed by library search using Mass Hunter METLIN Personal Compound Database and Library (PCDL) (Agilent Technologies, Santa Clara, USA) at MS/MS level.

Then, Principal component analysis (PCA) was used to evaluate reproducibility across measurements by checking the location of the QC samples on the PCA plots. After quality control, annotated compounds were used in the statistical analysis using MetaboAnalyst 4.0 [27]. Fold change and t-test, using false discovery rate (FDR) to correct for multiple testing, were performed to detect significant changes in certain metabolites between the pairwise samples over time. Orthogonal partial least squares discriminant analysis (OPLS-DA) model was built, and significant metabolites related to the differences between the pairwise samples were identified using the S-plot, comprising the combination of magnitude (covariance), with the effect and reliability (correlation) for the model variables concerning model component scores. Correlation analysis was performed using the difference between the values of the annotated features and the weighted pediatric Crohn’s disease activity index wPCDAI [28] scores (after EEN - before EEN) using Pearson r distance measurement.

## Results

An average of 8000 features were detected per sample from the total of samples analyzed, 6000–7000 features had formulas generated and 3000–4000 were putatively annotated. However, after a strict QC, 318 features were filtered with match score higher than 70 and are shown in the supplementary material containing their respective retention time and m/z (S1). PCA performed on those data showed clustering of all QCs together on obtained PCA models (Data not shown) which indicates the system’s stability and consistent performance throughout the analysis.

**Fig. 1:**
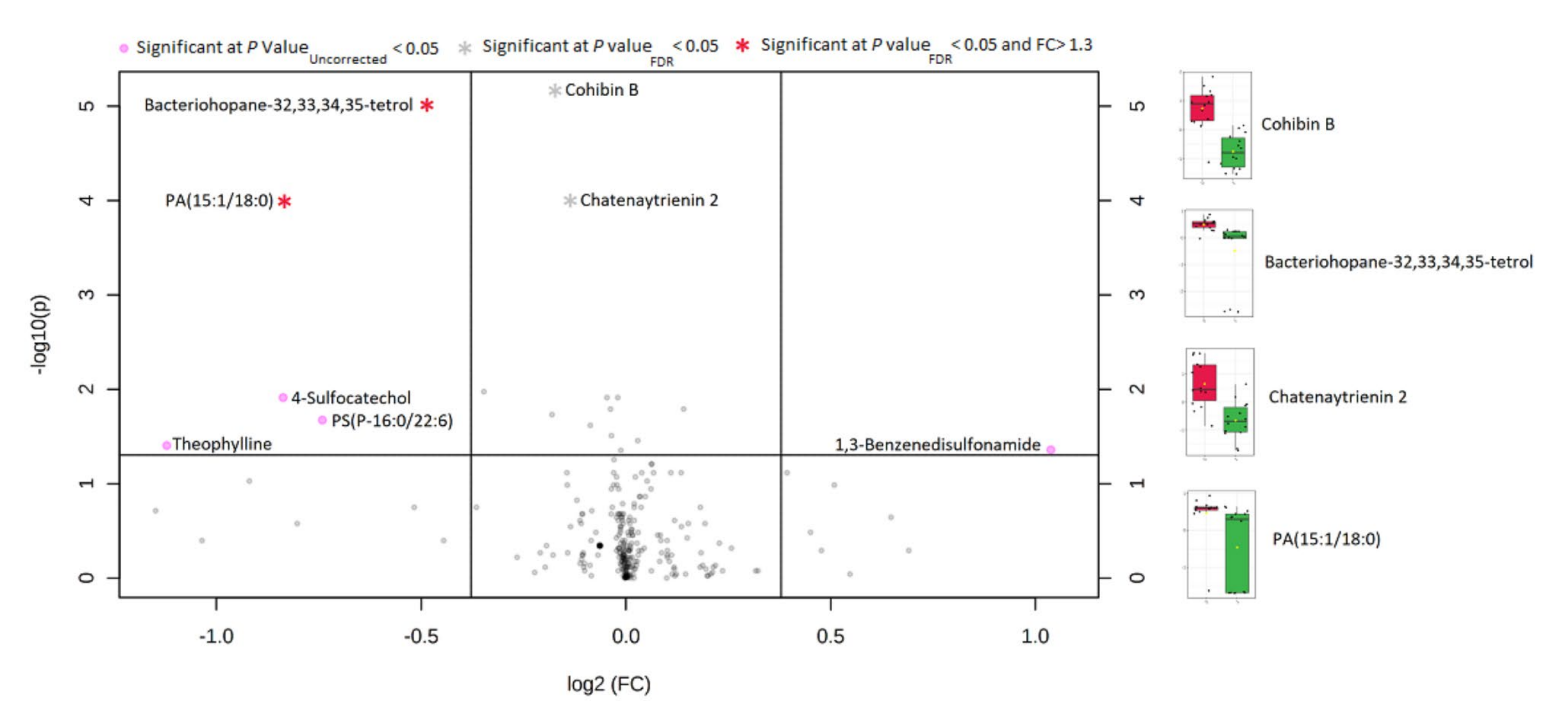
Volcano plot with log2 fold change (FC) in the x-axis and –log10 of p values on the y axis. The lines indicate FC > 1.3. Box whisker plot showing the interquartile range of the significant metabolites before (red) and after EEN treatment (green) * Entities significant after FDR correction for multiple testing

**Fig. 2:**
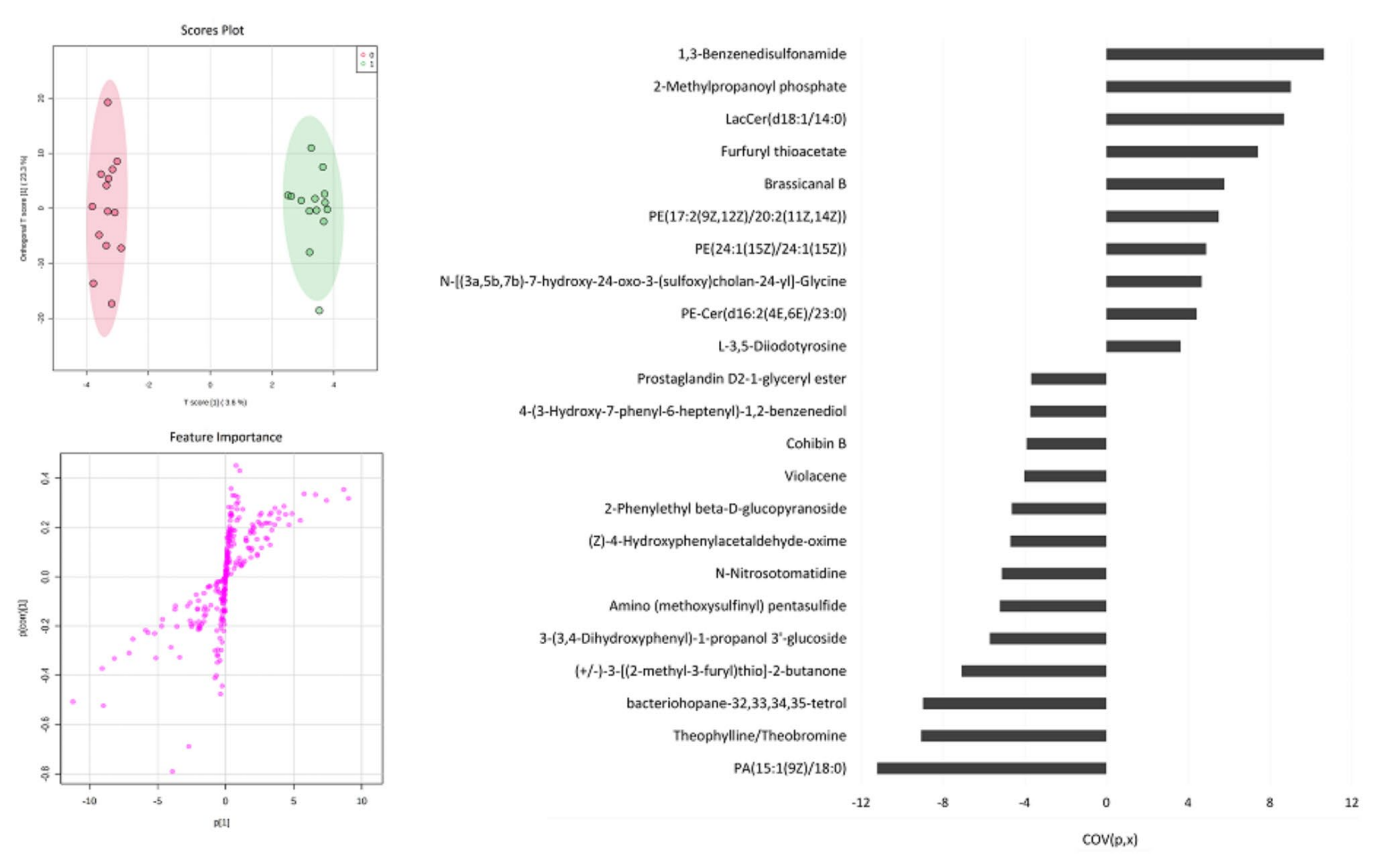
Scores plot and Splot from the Ortogonal partial least square discriminant analysis (OPLS DA) (Left) and bar chart with of the most important features in the model with cut off of 3.6

A volcano plot (Fig. 1) was built presenting the compounds filtered by pvalues and fold change (FC) and shows eight compounds found significant on the t-test with a p-value < 0.05 (Table 1). Albeit, after FDR correction, only 4 features remained significant at p < 0.05. Considering FC, with arbitrary cut-off of 1.3, two compounds (Bacteriohopane-32,33,34,35-tetrol, and PA(15:1/18:0)) were filtered as significant. Orthogonal PLS-DA model provided complete separation between the samples before and after EEN treatment. Figure 2 depicts the scores plot and the s-plot with features of importance showing the most significant compounds ordered by the covariance loading values obtained using an arbitrary cut-off value of 3.6, resulting in 10 annotated compounds with increased concentration after treatment and 13 compounds which decreased in concentration (Table 2).

**Table 1:** Results of univariate analysis using t-test

Feature	Change post-EEN therapy	p-value	p-value (FDR)
Bacteriohopane-32,33,34,35-tetrol	-	*	*
Cohibin B	-	*	*
PA(15:1/18:0)	-	*	*
Theophylline/Theobromine	-	*	na
4-Sulfocatechol	-	*	na
PS(P-16:0/22:6)	-	*	na
Chatenaytrienin 2	-	*	*
1,3-Benzenedisulfonamide	+	*	na

**Table 2:** List of the significant compounds using orthogonal PLS-DA ordered by the covariance loading values which increased or decreased post EEN therapy

Increased after EEN therapy	Decreased after EEN therapy
1,3-Benzenedisulfonamide	PA(15:1/18:0
2-Methylpropanoyl phosphate	Theophylline/Theobromine
LacCer(d18:1/14:0)	Bacteriohopane-32,33,34,35-tetrol
Furfuryl thioacetate	(+/-)-3-[(2-methyl-3-furyl)thio]-2-butanone
Brassicanal B	3-(3,4-Dihydroxyphenyl)-1-Propanol 3’-Glucoside
PE(17:2/20:2)	Amino (methoxysulfinyl) pentasulfide
PE(24:1/24:1)	N-Nitrosotomatidine
N-[(3a,5b,7b)-7-hydroxy-24-oxo-3-(sulfoxy)cholan-24-yl]-Glycine	(Z)-4-Hydroxyphenylacetaldehyde-oxime
PE-Cer(d16:2/23:0)	2-Phenylethyl beta-D-glucopyranoside
L-3,5-Diiodotyrosine	Violacene
	Cohibin B
	4-(3-Hydroxy-7-phenyl-6-heptenyl)-1,2-benzenediol
	Prostaglandin D2-1-glyceryl ester)

**Fig. 3:**
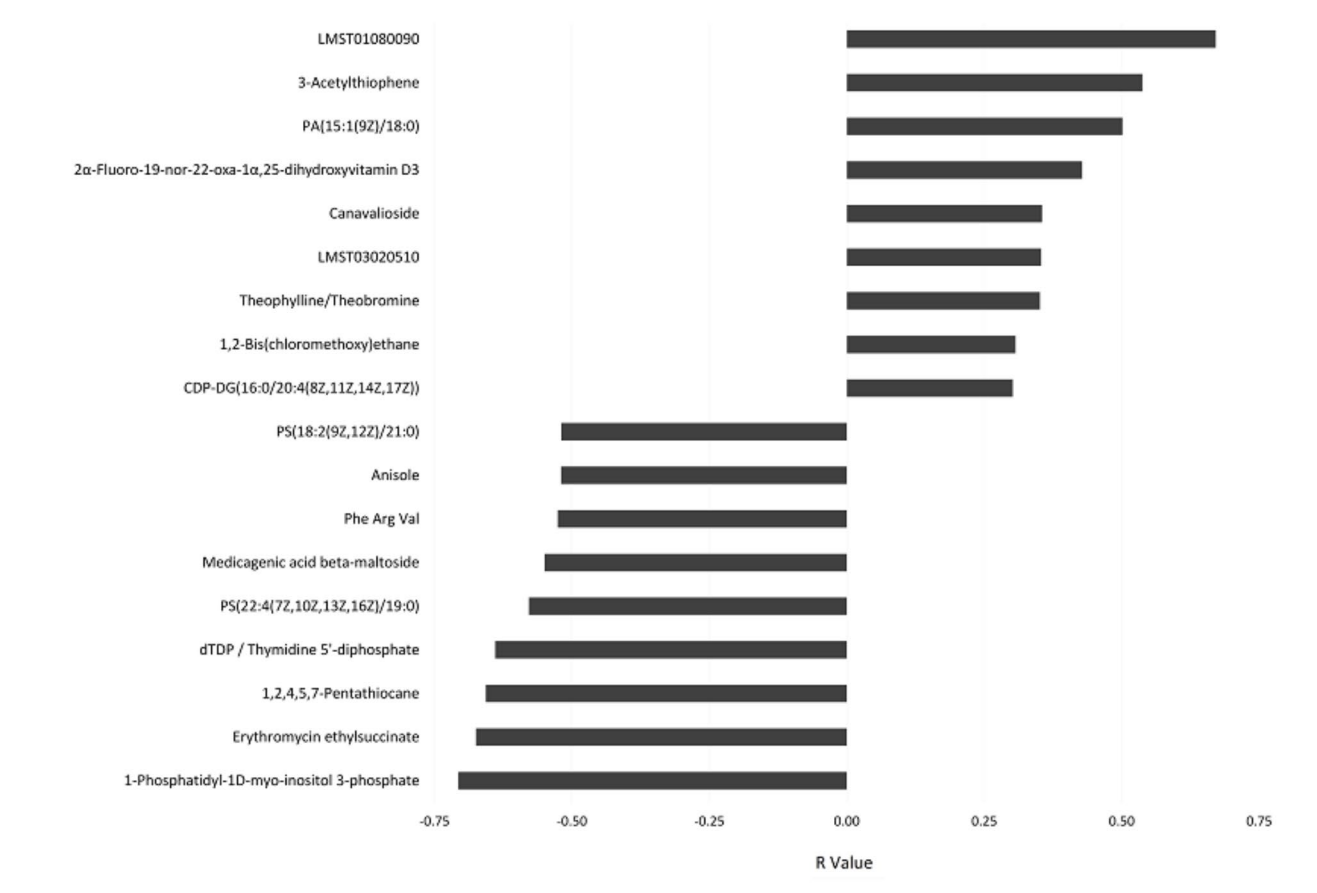
Bar chart showing features correlated with wPCDAI scores with Pearson correlation coefficient cut off of -0.5 for the negatively correlated features and 0.3 for the positive

As previously reported [15], disease activity measured by wPCDAI decreased significantly (p < 0.001) over the course of EEN treatment. The average score (± SD) was 48.8 ± 18.6 before start of EEN and decreased to 16.4 ± 10.1 after 3–4 weeks on EEN therapy. wPCDAI was developed as a score to stratify the severity of Crohn’s disease in pediatric patients with ranges between 0 to 100 with higher scores signifying more active disease. A score of < 12,5 is consistent remission, > 40 indicates moderate disease, and > 57,5 severe disease. A 17.5-point decrease in PCDAI is taken as evidence of small improvement and 37,5 as moderate improvement [28]. Correlation analysis was performed between the annotated compounds and the wPCDAI scores. The most significant compounds with R values < -0.5 for the negative correlation and > 0.3 for the positive are shown in Fig. 3. The following compounds were found negatively correlated with wPCDAI scores: 1-Phosphatidyl-1D-myo-inositol 3-phosphate; Erythromycin ethylsuccinate; 1,2,4,5,7-Pentathiocane; dTDP / Thymidine 5’-diphosphate; PS(22:4/19:0); Medicagenic acid beta-maltoside; Phe Arg Val; Anisole; and PS(18:2/21:0) with R values of -0.71, -0.67, -0.66, -0.64, -0.58, -0.55, -0.53, -0.52, -0.52, respectively). Conversely, we filtered 9 compounds positively correlated with wPCDAI: CDP-DG(16:0/20:4), 1,2-bis(Chloromethoxy)ethane; Theophylline/Theobromine; LMST03020510; Navalioside; 2α-Fluoro-19-nor-22-oxa-1α,25-dihydroxyvitamin D3; PA(15:1/18:0); 3-Acetylthiophene; and LMST01080090 ( R = 0.30, 0.31, 0.35, 0.35, 0.35, 0.43, 0.50, 0.54, and 0.67, respectively).

## Discussion

The use of EEN therapy clearly shows an impact on the plasma metabolome, with complete separation by orthogonal PLS-DA. Some features with a higher importance in the model appear related to the exclusion of a regular diet and they decreased most during EEN treatment. The compound annotated as Theophylline, for instance, presented the same match score as Theobromine, natural methylxanthines present in chocolate derivatives and beverages that are highly consumed by children and teenagers [29]. Other compounds related to diet that decreased during EEN therapy included: the food additive (+/-)-3-[(2-Methyl-3-furyl)thio]-2-Butanone [30]; organic compounds found in fruits: 3-(3,4-Dihydroxyphenyl)-1-Propanol 3’-Glucoside [31] and 2-Phenylethyl beta-D-glucopyranoside [32]; in tomatoes: N-Nitrosotomatidine [33] and other phytochemicals naturally occurring in fats and oils, green vegetables, herbs, and spices: (Methoxysulfinyl) Pentasulfide [34] and 4-(3-Hydroxy-7-phenyl-6-heptenyl)-1,2-benzenediol [35]. Violacene is a polyhalogenated monocyclic monoterpene [36] which is produced by diverse genera of bacterial strains which were isolated from various marine to freshwater and soil environments as well as marine algae and could probably reflect cessation of food intake [37]. A slight reduction shift was detected in Prostaglandin D2-1-glyceryl ester, a bioactive lipid involved in the endocannabinoid system with potential anti-inflammatory properties in vivo [38].

The compounds found elevated during EEN treatment in the multivariate analysis present two features annotated as phosphatidylethanolamine (PE), PE(24:1/24:1), and PE(17:2/20:2) and one ceramide phosphoethanolamine (PE-Cer): PE-Cer(d16:2/23:0). PE are estimated to comprise 15–25% of the total lipid content in mammalian cells and exert remarkable bio-activities [39]. In Gram-negative bacteria, PE comprises around 75% of the phospholipid cell envelop and are dynamic key compounds modulating metabolic activities [40].

Other elevated compounds were 3,5-Diiodo-L-tyrosine, involved in thyroid hormone synthesis [41] and LacCer(d18:1/14:0). Lactosylceramide (LacCer) was previously related with CD, however the role in the pathogenesis was unclear [42]. Another study indicated the potential of Lactosylceramide as a potential biomarker of inflammatory bowel disease in children [43]. LacCer is highly expressed in phagocytes and epithelial cells and may play an essential role in the human innate immune system, binding pathogenic microorganisms [44]. Furfuryl thioacetate, a naturally occurring aroma compound [45], and Furfuryl B, a secondary metabolite with antimicrobial activity produced by many plants source of edible vegetable oils [46] were also elevated. Two chemical entities appear with weighing importance in the OPLS-DA model, 2-Methylpropanoyl phosphate and 1,3-Benzenedisulfonamide; however, neither of them has been previously reported in the human metabolome. Interestingly, compounds containing the sulphonamide moiety present potential biological activities, such as carbonic anhydrase and COX-1/2 inhibition, as well as anti-inflammatory, and antitumor activities [47].

Some of the features with high importance in the multivariate model were found significant in the t-test and fold change, concomitantly. Bacteriohopane-32,33,34,35-tetrol, a bacterial metabolite, and biomarker of *Burkholderia, Pseudomonas, and Ralstonia spp* [48[, decreased during treatment. *Burkholderia spp* is a *Proteobacteria* known for causing dysfunction of GALT and gut microbiota in IBD [49], with the potential to invade intestinal epithelial cells [50]. An increase in the abundance of proteobacteria has been reported in IBD patients [51], in active or aggressive Crohn’s disease [52]. A decrease of Bacteriohopane-32,33,34,35-tetrol concentration after EEN treatment may indicate an effect on the gut microbiome with a decrease in *Burkholderia* and/or *Pseudomonas* population. Thus, the presence of elevated PE in plasma could be a marker of gram-negative bacterial cell membranes that underwent cell death.

Two other features annotated as Cohibin B and Chatenaytrienin 2 were significantly (p < 0.05) decreased with EEN treatment. Both compounds belong to the class of organic compounds known as annonaceous acetogenins, a class of natural compounds with a wide variety of biological activities and are powerful inhibitors of complex I (NADH : ubiquinone oxidoreductase) in mammalian and insect mitochondrial electron transport systems [53].

The most important metabolite in the OPLS-DA model was PA(15:1/18:0), also found significantly decreased in the t-test and fold change. PA is the simplest class of glycerophospholipids (GPL) present in virtually all organisms, from bacteria to higher plants. It is an intermediate in lipid membrane synthesis and storage and is also involved in many eukaryotic processes. Besides, PA influences membrane structure and interacts with diverse proteins due to its unique physicochemical properties in comparison to other GPL, thus acting as a lipid mediator in various signaling and cellular processes 54.

We correlated the metabolome with the patients’ wPCDAI scores to investigate what metabolites were associated with clinical improvement. The results show two metabolites in common with the list of features related to EEN treatment. There was a parallel decrease of PA(15:1/18:0), which correlated with wPCDAI scores, indicating that this metabolite could play a role in the CD pathophysiology. Another compound showing a similar trend was Theophylline/Theobromine reflecting exclusion of chocolate derivatives and beverages. These products were previously shown to modify the intestinal microbiome in a rat model, with a decrease in *E. coli; Bifidobacterium spp.; Streptococcus spp.; and Clostridium histolyticum-C* [55].

### Strengths and limitations

The strengths of our study are the use of a novel sensitive analytical methodology with the capability of detection of a wide variety of endogenous and exogenous compounds and inclusion of mostly treatment naïve patients. The limitations are the putative annotation of compounds, the small sample size, a limited number of two samples per patient, and the absence of dietary data at baseline, and the associations with hypothesis raising nature without providing evidence on causality.

## Conclusion

We report remarkable alterations in the plasma metabolic profile of pediatric CD patients treated with EEN. Markers reflecting the change of a mixed regular to a formula diet showed marked changes, including xenobiotics, such as food additives, other phytochemicals, and Theophylline/Theobromine which also correlated with a CD activity index. The decrease in the concentration of a biomarker of proteobacteria (Bacteriohopane-32,33,34,35-tetrol) and the increase in (PE) concentration, the main compound in cell membrane composition of gram-negative bacteria, together may indicate an alteration in potential pathogenic bacteria populations and metabolism. Thus, metabolomics provides an opportunity to characterize the molecular mechanisms of dietary factors triggering CD activity and unravel the mechanisms of action of EEN.

## Electronic supplementary material

Below is the link to the electronic supplementary material.

Supplementary Material 1

## Data Availability

The data underlying this article will be shared at reasonable request to the corresponding author.
